# Representation of the verb's argument-structure in the human brain

**DOI:** 10.1186/1471-2202-9-69

**Published:** 2008-07-21

**Authors:** Ramin Assadollahi, Brigitte S Rockstroh

**Affiliations:** 1 Department of Psychology, University of Konstanz, 78457 Konstanz, Germany; 2 ExB Communication Systems GmbH, 80333 Munich, Germany

## Abstract

**Background:**

A verb's argument structure defines the number and relationships of participants needed for a complete event. One-argument (intransitive) verbs require only a subject to make a complete sentence, while two- and three-argument verbs (transitives and ditransitives) normally take direct and indirect objects. Cortical responses to verbs embedded into sentences (correct or with syntactic violations) indicate the processing of the verb's argument structure in the human brain. The two experiments of the present study examined whether and how this processing is reflected in distinct spatio-temporal cortical response patterns to isolated verbs and/or verbs presented in minimal context.

**Results:**

The magnetoencephalogram was recorded while 22 native German-speaking adults saw 130 German verbs, presented one at a time for 150 ms each in experiment 1. Verb-evoked electromagnetic responses at 250 – 300 ms after stimulus onset, analyzed in source space, were higher in the left middle temporal gyrus for verbs that take only one argument, relative to two- and three-argument verbs. In experiment 2, the same verbs (presented in different order) were preceded by a proper name specifying the subject of the verb. This produced additional activation between 350 and 450 ms in or near the left inferior frontal gyrus, activity being larger and peaking earlier for one-argument verbs that required no further arguments to form a complete sentence.

**Conclusion:**

Localization of sources of activity suggests that the activation in temporal and frontal regions varies with the degree by which representations of an event as a part of the verbs' semantics are completed during parsing.

## Background

Most verbs describe events with one or more participants [1]. The verb's argument structure defines the number and relationships of participants needed for a complete event. For "Peter gives Jim a book", linguistic theorizing [2] would ascribe participants three thematic roles: the agent (Peter), the recipient (Jim) and the theme (the book). The entry in the mental lexicon for a verb like "give" must incorporate such information in addition to phonetic and orthographic information.

The cortical processing of argument structures has been investigated mostly in designs employing entire sentences, wh-questions (that is questions starting with 'what', 'which', who' or else), or sentences including syntactic or semantic violations [3-6,2]. Imaging studies suggest that the middle temporal gyrus (MTG) and the inferior frontal gyrus (IFG, BA 45/47) of the left hemisphere [7-9] play a crucial role in this processing. In particular, the left IFG (BA 44/45) has been shown to be active when grammatically complex sentences that required working memory resources [10,11], and when argument hierarchies were processed [3]. Even if words were presented in the grammatically correct order in one, and out of order in another condition, activation of the left IFG and MTG was more pronounced to words in correct sentences [12]. Complementing this research on sentence processing, the present study examined whether the verb's argument structure was already retrieved with the verb itself. For this purpose, electromagnetic brain responses to verbs of different argument structure, presented in isolation, were assessed. As an alternative, the information about argument structure might be retrieved only in context, so that the brain response discloses the relation between argument structures and their fillers. This option was examined in a second experiment by measuring the electromagnetic brain responses to verbs that varied in their argument structure and were presented in a minimal syntactic context. Compared to full-sentence designs and isolated verb presentation, a minimal context should prevent influences of other words and structures within a sentence, and hence disclose the extent to which relatively small differences in the lexical entry can be traced in the brain response.

Retrieval of the argument structure from the verb itself has been suggested by behavioral studies, which reported faster responses to two-compared to three-argument verbs [13]. But it has also been argued that context is required to activate syntactic processing [3,6]. This, too, is supported by faster responses to verbs related to nouns (i.e. matching one of the arguments) relative to unrelated verbs [14], by faster responses whenever two-compared to three-argument verbs had to be integrated into sentences (Ahrens, 2003), and by faster responses to words following verbs with one compared to three participant roles [15,16]. An impact of context can also be concluded from distinct activation including the left IFG after sentences [11].

For the present study, we selected verbs with one, two, or three obligatory arguments (e.g. 'snore', 'meet', or 'give'). If isolated verbs activated the processing of their argument structure, more pronounced activation by the more complex relative to the simple argument structure were to be expected mainly in left anterior/middle temporal areas. This hypothesis was examined in a first experiment, in which isolated verbs with different argument structure were presented visually. The second experiment served to explore the impact of the verb's context. If presentation of the verb was sufficient for the retrieval of its argument structure, and if context was needed to activate syntactic processing, would the minimal context of just one noun (a proper name) be sufficient to activate this processing? If so, name-verb pairs with different argument structures should evoke different cortical response patterns. We hypothesized that the minimal context should start the evaluation automatically, whether or not the verb completed a grammatically correct sentence. Only for one-argument verbs, the name completes a sentence. If a minimal context would activate the same processing as an entire sentence, left IFG activation distinguishing the conditions (i.e. name-verb pairs with one-argument versus two- and three-argument verbs) was to be expected.

## Results

### Experiment 1

**Figure 1:**
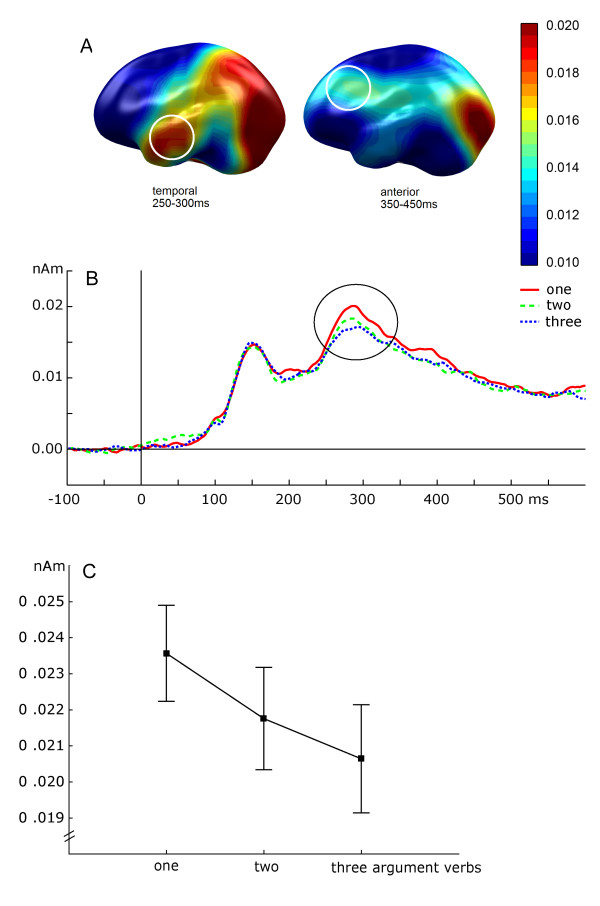
A: Topographical distribution of cortical activation (left-hemispheric view) in the source space (Minimum Norm Estimates, MNE, in nanoAmperemeter, nAm) for verbs presented in isolation, averaged across subjects. MNE were projected onto a standard brain following [28,29]. In the time window 250–300 ms (left graph) after stimulus onset, most prominent activation occurred in temporal areas; activation in the frontal areas was weaker and later (350–450 ms, right graph). B: Time course of activation for verbs presented in isolation averaged across subjects separately for the three verb categories (with one, two and three arguments) in the left temporal area. C: Group mean and standard error of activity (ordinate: MNE amplitude in nAm) for isolated verbs (abscissa: verbs with either one, two or three arguments) at 250–300 ms.

Around 250–300 ms, one-argument verbs led to the strongest activation in the left temporal lobe, while three-argument verbs produced the weakest activation, and activation by two-argument verbs were in between (linear trend: F(1,21) = 6.2, p = 0.023; Figure 1). Between 350 and 450 ms activity did not vary between verbs (F<1).

### Experiment 2

Similar to experiment 1, activity around 250–300 ms in the left temporal lobe was most pronounced to one-argument verbs. However, the linear trend was not significant (p = 0.1). (Across the two experiments, the linear trend was significant with F(1,21) = 7.88, p = 0.01).

**Figure 2:**
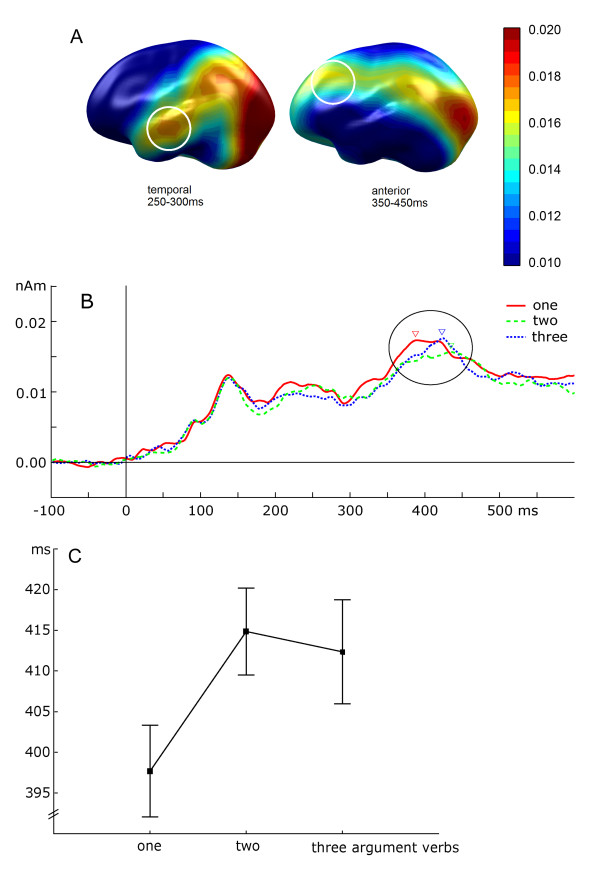
A: Topographical distribution of cortical activation (left-hemispheric view) in the source space (MNE in nAm) for verbs presented in minimal context. Anatomical projection as in Figure 1. In contrast to experiment 1, the early activation (250–300 ms, left) in the temporal region is weaker, whereas later activation (350–450 ms, right) in the more frontal region is stronger. B: Time course of activation for verbs presented together with a noun averaged across subjects separately for the three verb categories (with one, two, and three arguments). C: Group mean and standard error of the latencies (ordinate: in msec) for name-verb pairs with verbs including either one, two, or three arguments (abscissa).

Between 350 and 450 ms, activity varied in amplitude and latency in the region of the inferior frontal gyrus when a name preceded the verb (Figure 2). A linear trend confirmed larger amplitudes for one- than for two- and three-argument verbs (F(1,21) = 7.9, p = 0.01) when verbs were presented in minimal syntactic contexts of names. Moreover, activity peaked significantly earlier when the name-verb pair formed a complete (see Figure 2C), and thus grammatically correct sentence (mean peak latency for one-argument verbs: 397 ms) compared to pairs forming incomplete sentences (mean peak latencies for verbs with two obligatory arguments: 414 ms, with three arguments: 412 ms; main effect: F(2,42) = 5.2, p = 0.009, post-hoc comparison one- vs two-argument verbs: F(1,21) = 9.4, p = 0.005; for one- versus three-argument verbs: F(1,21) = 5.4, p = 0.029; for two- vs three-argument verbs: n.s.).

## Discussion

Two results of the present study seem noteworthy: (1) Verbs of different argument structure differentially activate cortical areas in the left middle temporal lobe, the distinct processing being reflected in electromagnetic response patterns as early as 250 ms after stimulus onset. (2) Whenever verbs were presented in combination with a name, and were thus processed within a minimal context, an additional later (350–400 ms) and more anterior activation in the left hemisphere showed the same differentiation by the verb's argument structure as the earlier one. From the projection of MNE onto a standard anatomical brain (as in Figures 1 and 2), we may assume that the first activation involves the middle temporal gyrus, while the second activation might involve areas like the IFG and Broca's area. Thus, these structures may participate in this analysis of argument structure in syntax processing. Further support of this anatomical relationship and the significance of the IFG is drawn from recent imaging (fMRI) results obtained from a similar experimental design [Assadollahi et al., submitted], which disclosed the IFG and the left MTG as primary areas distinguishing conditions.

Both results suggest that the processing of a verb's argument structure is linked to the verb itself and does not require a complete sentence. Moreover, the differential activation that is even evoked by the minimal context of a name suggests that a major impact of working memory can be ruled out [5].

Activation in the middle temporal gyrus followed a linear trend (when averaged across experiments). This, too, indicates the activation of argument structure by verbs independent of any participants. McRae and coworker [33] found a priming effect even under conditions when the subject (agent-filler) followed the verb, which is a rare construction in every day English. Thus, a verb can indeed activate the argument structure even if the filler is not provided. In the active anterior region (presumably including the IFG), this linear trend was only evident when verbs were preceded by names. This suggests that the IFG is involved in keeping representations active (the names) and in integrating them with further incoming representations (the verbs). In line with this hypothesis McDermott and colleagues [34] point out that Broca's area is not only active during processing of syntax but also involved in semantic tasks. There is also evidence that larger parts of the left inferior frontal gyrus are involved in syntactic processing [10,11,35-39]. Activation during semantic processing has consistently been found in left inferior frontal areas [40-48]. Specifically, a region in the anterior and ventral aspect of the inferior frontal gyrus (IFG, approximate BA47/10) has been identified as contributing to semantic processing, in addition to the left middle temporal cortex [50,51] (which was also active in the present study). Thus, we may conclude that the IFG projects back to the temporal lobe to keep representations active. Such a structure would allow for lexical items to interact when coming in sequentially: The activation of typical fillers (subjects or obligatory objects) is supposed to facilitate on-line language processing. Verbs have been demonstrated to prime typical fillers of agent, patient and instrument roles and vice versa [14,33]. Sentential information (a subject and obligatory objects), structurally crucial to a verb (defined by its argument structure), facilitates integration of the verb into the sentence. As a consequence, reading is accelerated when thematic roles are saturated (e.g. the subject was provided) during comprehension [50,52]. In the present study, processing speed (as indicated by the time of the peak activation) distinguished the automatic evaluation of verbs requiring one argument as grammatically completing a sentence from the evaluation of verbs requiring two or three arguments as leaving an incomplete, grammatically incorrect sentence. The interplay between IFG and middle temporal gyrus may be the biological substrate for these priming effects.

The assumption that sequential processing of lexical items (like verbs) is reflected in distinct spatio-temporal brain activation patterns fits with theoretical models: For instance, Rappaport and colleagues [53] assume that a verb activates an intermediate representation, the semantic class. The semantic class provides the list of lexical conceptual structures and associated argument structures defining different argument structures and roles. The activation of the semantic class may involve allocation of memory for the upcoming syntactic structure of the sentence. It is conceivable that the amount of memory allocation depends on the complexity of the expected sentence and, hence, on the verb's argument structure. It has been suggested that the number of thematic roles is related to the processing load which is required for the integration of single words into sentences [15], and that the inferior frontal gyrus (IFG) is involved in the transient storage of information during parsing [10,11,15,53]. Stromswold and colleagues [11] reported increased regional cerebral blood flow (rCBF) in Broca's area, particularly in the pars opercularis, when subjects judged the semantic plausibility of syntactically more relative to less complex sentences. This suggests that the activation of Broca's area may vary with the complexity of the sentence.

**Figure 3:**
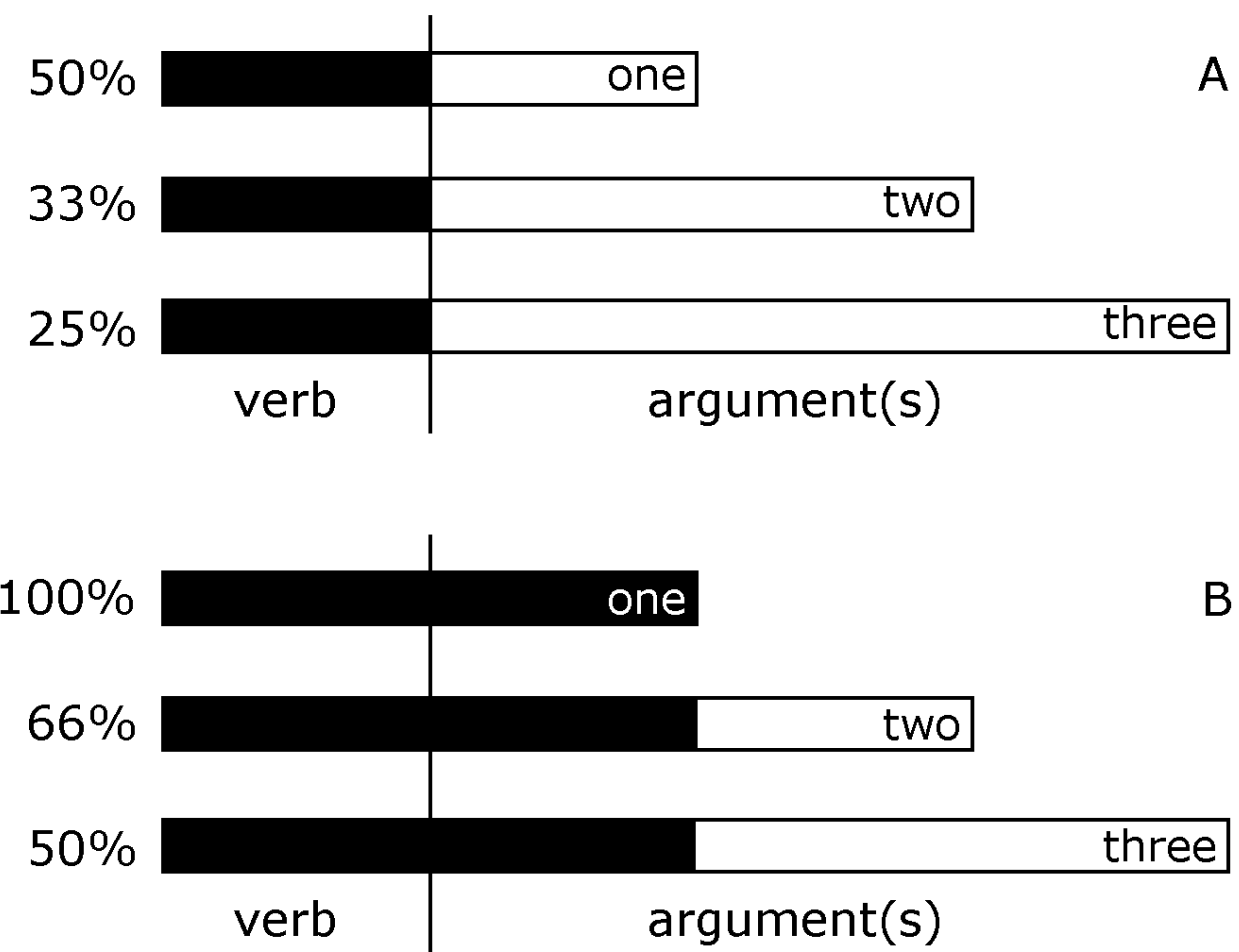
Schematic concept how the integration of arguments into representations of events could lead to different brain activation. Bars extending to the left represent the completeness of the representation; each group of bars represents the different argument structures. Incoming words fill the slots of the scene representations (black bars).

In contrast to previous evidence and our expectation, more verb arguments did not provoke more activation. Instead, decreasing activation with increasing complexity in argument structure was found. A similar result was also obtained in our recent fMRI study using different stimuli and a different experimental setup. This result may indicate that activation reflects integration rather than processing demands imposed by the verb. Within the composition of words (including verbs) into a sentence, the completeness of the compositional representation of words may vary across the parsing process, and may be accompanied by a sequence of activation (see illustration in Figure 3). Before the first word is presented, sentence processing (or verb retrieval) is 0% complete, and activation has not started. Activation starts with word/verb presentation, with retrieval of the verb describing the situation being of highest impact. While the recognition of simple events is 50% complete (subject missing), the recognition of complex events may be less complete (33% for two place verbs, 25% for three place verbs, Figure 3A). Thus, we may assume that the representation of an event is more likely activated by a verb with few arguments. Finally, event representation composition is complete when all parts are given. This is the case for one-argument verbs when they are presented together with names (Figure 3B). Although speculative, this model would explain the differential activation in the temporal lobe as reflecting the degree to which event retrieval is complete, with activity being higher for one- than for three-argument verbs.

Semantic complexity, examined for eventive and stative verbs, has been found to affect processing time [54]. Completion of the event's representation may have been reflected by the (earlier) peak of activity in IFG. The parser may be waiting for further input in the other cases. Behavioral studies have shown that appropriate versus inappropriate syntactic context can affect a target word's naming latency or lexical decision time or both [55-59]. Similar timing can be reflected prior to overt reaction, i.e. in the latency of peak amplitudes of the brain response to the word in context. Many studies on word frequency effects reported shorter peak latencies of brain responses corresponding to shorter reaction times [60-62]. In the present study, the earlier amplitude in correct phrases may be a signature of a sentence's end similar to the EEG findings of a late positivity following the last word of a grammatical sentence [63,64].

## Conclusion

In sum, the present results suggest that sub-representations of entries in the mental lexicon are processed along a dimension of complexity. This processing evolves over time and occurs in the brain along a posterior-anterior axis: Whereas recognition of the visual items activated posterior brain areas around 150 ms after stimulus onset without distinct processing of item features, the argument structure inherent in a verb was automatically distinguished at some 250 ms latency in the middle temporal lobe, followed by the automatic appraisal of the grammaticality of the verb presented in minimal context at some 350 ms latency in more frontal areas, presumably the IFG.

## Methods

### Sample and study design

The study protocol conformed to the Code of Ethics following the Declaration of Helsinki 1964 and was approved by the Ethics Committee of the University of Konstanz. The experimental sample included 22 healthy right-handed subjects (native speakers of German, 11 female, mean age: 24 years) naïve to the experimental purpose. Subjects were informed about the experimental procedures and the magnetoencephalographic (MEG) measurement and gave written consent. Subjects were instructed that words would be presented visually during the MEG measurement, that they should read each word carefully, and that they should fixate a cross on the screen during stimulus-free intervals. They were further instructed that the stimulus series contained verbs and nouns and that they should press a button only in response to the nouns. (This task was introduced to ensure sustained attention, without being relevant to the experimental question.) After the experiments, an interview evaluated whether subjects were naïve with respect to the three different verb argument structures. Indeed, none of the subjects noticed that the presented verbs differed in argument structures. An additional sample of 10 student volunteers was recruited for the pilot study, which served the purpose of stimulus selection (see below). All subjects received a financial bonus for participation.

### Material and tasks

In the pre-experimental pilot study, 10 student volunteers were asked to generate a sentence to each of 600 German verbs that were pre-selected from the CELEX-database [17] and presented in third person singular present active form with different argument structures. Verbs were selected to be unambiguous and to have the lowest possible number of different argument structures [13,18]. Non-obligatory adjuncts referring to time or space were not considered. A verb was approved for the experimental stimulus set when more than 70% of the generated sentences included the argument structure of the central sense [19]. For each of the three categories of one-, two-, and three-argument verbs 130 verbs were selected. Categories were matched for length (mean: 7.8 letters, c.f. [20,21] and frequency (mean: 2.3 per million words [17]).

In both experiments, the stimulus series comprised 130 one-, 130 two- and 130 three-argument verbs presented in pseudo-random order. Verbs were presented in white upper-case letters (maximum word size 9 × 3 cm) on a black background at a distance of 1.4 m for 150 ms each with inter-stimulus intervals varying between 1200 and 2000 ms. All verbs were shown only once per condition in order to avoid repetition effects [22]. Nouns, derived from the verbs, but identifiable as nouns, were interspersed with 10% probability in the stimulus series, and subjects were asked to press a button whenever they identified a noun in order to ensure sustained attention and access to the mental lexicon. In experiment 1, verbs were presented in isolation. In experiment 2, the same stimuli were presented in a different order and, while each verb was preceded by a name, which appeared 500 ms before each verb for 150 ms. The 500-ms inter-stimulus interval should allow to establish a Conceptual Short Term Memory for a word [23,24]. For the response task, new nouns differing from those of experiment 1 were created.

### MEG data acquisition and analysis

Electromagnetic signals were recorded with a 148-channel whole-head magnetometer (Magnes 2500 WH, 4D NeuroImaging Inc., San Diego) using a 0.1–100 Hz band-pass filter and sampled at a rate of 508 Hz. Vertical and horizontal EOG (electrooculogram) were recorded for control of artifacts. Subjects lay horizontally and stimuli were projected onto a screen on the ceiling. It was ensured that the MEG sensor (precisely the lower rim of the dewar) was 90° to the floor and subjects' heads were positioned horizontally. In this way the head rotation error induced by a subject's head position was minimized. After external global noise subtraction, continuous MEG data were segmented into 900-ms epochs (including 100 ms before and 800 ms after stimulus onset). Artifact-contaminated epochs (EOG level > 100 mV, MEG level > 5 pT, button press) were excluded. This resulted on average in 100 traces (of the total 130 stimuli) per subject and condition suitable for analysis. For each subject and for each verb category, stimulus-locked evoked magnetic fields (EMFs) were determined relative to the 100-ms pre-stimulus baseline.

For the average EMFs, cortical sources were determined using the Minimum Norm Estimate (MNE) based on a spherical volume conductor [25,26]. The MNE [25,27,28] represents an inverse method to reconstruct the topography of the primary current underlying a magnetic field distribution [29] within acceptable residual variance (here: <5%). Pseudo-inverse matrices were regularized (Tikhonov-Phillips, λ = 0.01). Following Hauk and co-workers [25], cortical activity was estimated in a three-dimensional source space consisting of four concentric spheres, with the outer shell being fitted to the individual head-shape of the subjects (4-D Neuroimaging software). According to Sarvas [30], the radius of the head has no effect on the estimated magnetic field generated by primary currents in a spherically symmetric volume conductor. For the present analysis, the head radius was estimated to be 10 cm. We report MNE for the shell at 80% radius, which roughly corresponds to the cortex in the brain. Following Hauk, this radius most closely resembles cortical activity, which is the aspect of the data we were focusing on. On this sphere 197 equidistant locations of dipoles each represented by two tangential orientations were assumed. The dipole strength (as represented in the figures, was computed as root mean square of the two tangential dipoles at each location. For visualization MNE were projected onto a standard brain following Moratti and Junghöfer [31,32].

For brain activation analysis, distributed source activity (MNE), averaged over 50-ms windows, was screened for activity peaks and differences between the verb's argument structures. Three peaks of activity were evident: an early, bilateral temporo-occipital activation around 150 ms after stimulus onset, a left-hemispheric temporal activation around 250–300 ms, and a left anterior activation peak between 350 and 450 ms. Since no variation with experimental manipulation (verb's argument structure) was found for the first peak, this component was discarded from further analysis. For the 250–300 ms interval, a Region of Interest (ROI) was defined as the average strength of 6 dipoles over the left temporal lobe. Differences between verb categories (one-. two-, and three-argument verbs) were verified for the average MNE amplitude over the ROI and for the peak amplitude within each ROI by means of linear trends and analyses of variance (ANOVA). F-tests were used to further investigate significant main effects.

## Abbreviations

BA: Brodman's area; EMF: electromagnetic field; EOG: electrooculogram; fMRI: functional magnetic resonance imaging; IFG: inferior frontal gyrus; MEG: magnetoencephalogram; MNE: minimum norm estimate; MTG: middle temporal gyrusp; pT: pico Tesla; ROI: region of interest.

## Competing interests

The authors declare that they have no competing interests.

## Authors' contributions

RA proposed the general research question to investigate three argument structures, accomplished data collection and analysis. RA and BR designed the experiments and wrote the manuscript.
